# Lymphome non-hodgkinien pulmonaire primitif: à propos d’un cas

**Published:** 2012-03-24

**Authors:** Aziz Ouarssani, Fouad Atoini, Fatima Ait Lhou, Mustapha Idrissi Rguibi

**Affiliations:** 1 Service de pneumologie, Hôpital Militaire Moulay Ismail, Meknès, Maroc; 2 Service de chirurgie thoracique, Hôpital Militaire Moulay Ismail, Meknès, Maroc

**Keywords:** Lymphome pulmonaire, biopsie, immunohistochimie, phénotype B, opacités, radiographie du poumon, Maroc

## Abstract

Le lymphome pulmonaire primitif (LPP) est rare, représente 3 à 4% des lymphomes malin non hodgkinien (LMNH) extra ganglionnaire; moins de 1% des LMNH et seulement 0.5 à 1% des tumeurs malignes primitives du poumon. Les lymphomes non hodgkiniens de phénotypes B de haut grade de malignité représentent 11 à 19% des cas de lymphome pulmonaire primitif. Nous rapportons l’observation d’un patient âgé de 47 ans, sans antécédents pathologiques particuliers; hospitalisé pour une toux traînante associée à des opacités multiples excavées et bilatérales. C’est la ponction transparietale scannoguidée avec étude anatomopathologique qui confirme le diagnostic de lymphome pulmonaire primitif de haut grade de malignité de phénotype B. Les lymphomes pulmonaires primitifs sont rares; leur symptomatologie clinique et radiologique est non spécifique et c’est l’étude anatomopathologique et immuno-histo-chimique qui confirme le diagnostic.

## Introduction

Le poumon peut être le siège d’une atteinte lymphomateuse dans trois situations [1,2]: 1) Par dissémination hématogène à partir d’un lymphome ganglionnaire non hodgkinien; 2) Par envahissement par continuité à partir d’une localisation hilaire ou médiastinale d’un lymphome ganglionnaire; 3) enfin par une atteinte pulmonaire primitive : c’est les lymphomes pulmonaires primitifs qui sont définis comme des proliférations lymphoïdes clonales atteignant un ou les deux poumons (parenchyme et ou bronche) sans atteinte extra pulmonaire mise en évidence au moment du diagnostic et dans les trois mois qui suivent.

Les lymphomes pulmonaires primitifs sont des tumeurs très rare; ils représentent 3à 4 % des lymphomes non hodgkinien extra ganglionnaire; moins de 1% des lymphomes non hodgkinien et seulement 0,5%à1% des tumeurs malignes primitives du poumon; ils regroupent actuellement [2]: 1) Les lymphomes pulmonaires primitifs de phénotypes B de bas grade de malignité les plus fréquentes 58à 87% des cas et dans 90% des cas il s’agit d’un type MALT; 2) Les lymphomes pulmonaires primitifs de phénotype B de haut grade de malignité; 3) La granulomatose lymphomatoïde beaucoup plus rare.

## Patient et observation

**Figure 1:**
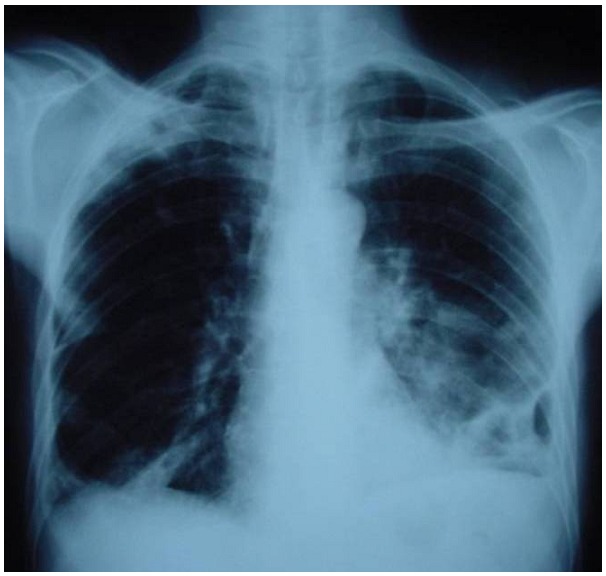
Radiographie thoracique de face objectivant de multiples opacités excavées bilatérales

Monsieur HL; âgé de 47ans; sans antécédents pathologiques notables, et sans habitudes toxiques, hospitalisé au service de pneumologie pour une toux productive ramenant des expectorations purulentes évoluant depuis 2mois. L’examen trouvait un patient en assez bon état général; apyrétique, l’examen pleuro pulmonaire trouvait des râles crépitant bilatérales à l’auscultation, les aires ganglionnaires étaient libres, la radiographie thoracique de face objectivait des opacités excavées bilatérales (Figure 1).

**Figure 2:**
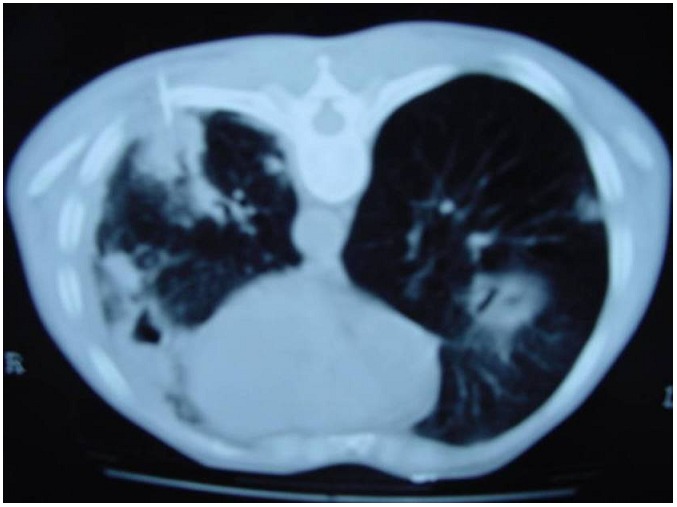
Scanner thoracique montrant des opacités excavées avec ponction transpariétale

Le scanner thoracique confirmait l’existence de multiples lésions pulmonaires excavées bilatérales celle du coté gauche est fistulisée dans la plèvre entraînant un pneumothorax en regard (Figure 2).

La biologie objectivait une vitesse de sédimentation à 65mm à la 1ere heure; l’hémogramme et l’ionogramme sanguin étaient normales, la sérologie VIH était négative; les recherches du bacille de Koch étaient négatives à l’examen directe; l’intradermoréaction a la tuberculine à 10unités était à 6mm. La fibroscopie bronchique était normale; et la recherche de germes banals; de mycoses, du bacille de koch et de cellules malignes était négative dans le liquide d’aspiration bronchique.

**Figure 3:**
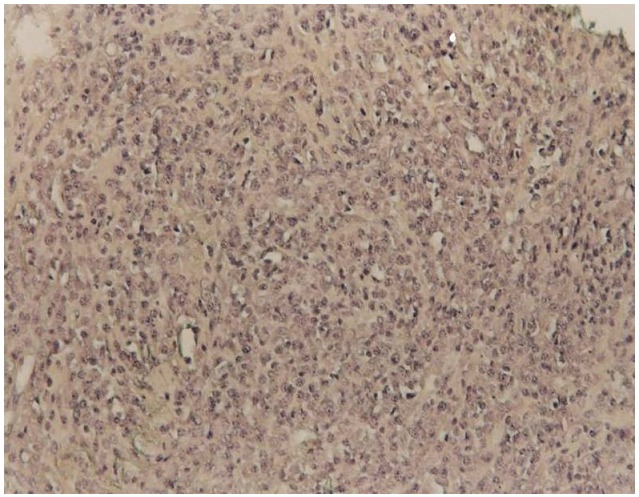
Etude anatomopathologique avec de petites cellules lymphoïdes de distribution diffuses

**Figure 4:**
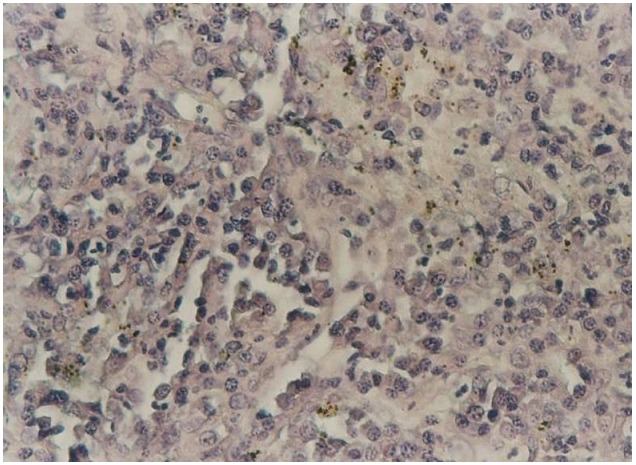
Etude immunohistochimique montrant que le marqueur CD20 est positif

Une ponction transpariétale scannoguidée a été effectuée (Figure 2), l’étude anatomopathologique et immunohistochimique confirmait le diagnostic de lymphome malin non hodgkinien de phénotype B de haut grade de malignité (Figure 3 et Figure 4).

Un bilan d’extension prethérapeutique a été effectué; le scanner abdominal était normal, la biopsie osteomedullaire ne montrait pas d’envahissement médullaire, la fibroscopie œsogastroduodénale était normale; l’examen ophtalmologique et oto-rhino-laryngologique étaient normaux.

Le patient a bénéficié d’une chimiothérapie type CHOP cyclophosphamide, doxorubicine, oncovin, prednisone. Une semaine après la 5éme cure de chimiothérapie, le patient fut admis en réanimation pour un syndrome de détresse respiratoire aigue sévère et décédait dans un tableau de choc septique grave.

## Discussion

Les lymphomes non hodgkinien B de haut grade représentent 11 à 19% des cas de lymphome pulmonaire primitif [1]. Ils surviennent souvent sur un terrain particulier : transplanté d’organe solide (cœur/ poumon) recevant de la cyclosporine A ou de l’OKT3, sujet infecté par le virus de l’immunodéficience humaine VIH ou présentant un syndrome de Gougerot-sjogren; ou chez des patients avec séquelles pleurales de collapsothérapie.le rôle du virus de l’Epstein Barr (EBV) a été mis en cause [2].

L’âge de survenue est en moyenne de 60 ans (30 à 80 ans), les signes cliniques sont de type respiratoire avec parfois fièvre et amaigrissement, l’aspect radiologique est celui d’une masse pulmonaire, une atélectasie, un épanchement pleural est le plus souvent associé. Des opacités multiples excavées sont fréquemment retrouvées chez les patients VIH positif, notre patient avait des opacités excavées avec une sérologie VIH négative [3,4,7,8].

L’endoscopie bronchique est souvent anormale, avec un bourgeon ou une sténose infiltrative d’allure tumorale, le diagnostic histologique est en règle facile par biopsies bronchiques, transbronchiques ou transpariétales avec présence de cellules lymphoïdes à caractère blastique et à forte activité mitotique.

Il s’agit le plus souvent de lymphome de type immunoblastique et centroblastique, le diagnostic différentiel est limité grâce à l’immunohistochimie on éliminera un carcinome, un mélanome, ou un sarcome [5]. Chez notre patient le diagnostic a été obtenu par ponction transpariétale scannoguidée.

Le bilan pré-thérapeutique comporte un scanner thoracoabdominal, une biopsie ostéomedullaire montrant des signes d’envahissement dans 20à 30% des cas un examen ophtalmologique et ORL une fibroscopie œsogastroduodénale et une coloscopie, une électrophorèse et une immunoélectrophorèse sérique. Les traitements utilisés sont la chirurgie en cas de lésion localisée, la chimiothérapie en cas d’atteinte bilatérale ou extra pulmonaire; de rechute ou de progression. La radiothérapie est peu utilisée L’efficacité respective de ces traitements ne peut être analysée du fait de l’absence de séries comparatives Les poly chimiothérapie de type CHOP : cyclophosphamide, doxorubicine, oncovin prednisone n’ont pas montré de supériorité thérapeutique vis-à-vis d’une monochimiothérapie à base de Chloraminophéne de cyclophosphamide, d’azathioprine ou de corticoïde [6].

Notre patient a bénéficié d’une chimiothérapie de type CHOP. Le pronostic est moins bon que les lymphomes de bas grade de malignité, la survie est de 8 à 10 ans mais cette survie est bien inférieur si le terrain est particulier: VIH, greffé.

## Conclusion

Les lymphomes pulmonaires primitifs B de haut grade de malignité sont rare, surviennent souvent sur un terrain particulier, bien que leur diagnostic est facile mais leur prise en charge thérapeutique reste encore non codifiée et les rechutes locales ou à distance sont plus fréquentes.
